# PS-16 - APPLYING HEALTH EQUITY ASSESSMENT TO ACUTE HEALTH PROTECTION RESPONSES IN SOUTH EAST ENGLAND

**DOI:** 10.1093/pubmed/fdag046.063

**Published:** 2026-07-09

**Authors:** Dr Catherine Alber, Jack Tomkinson, Dr David Roberts

**Affiliations:** UK Health Security Agency South East; UK Health Security Agency South East; UK Health Security Agency South East

## Abstract

**Background:**

Communicable disease and non-infectious environmental health protection hazards are strongly associated with inequalities in both risk and severity, and universal health interventions may not benefit all groups equally. UK Health Security Agency Health Protection Teams (HPTs) provide specialist public health advice to local stakeholders and the public; however, the extent to which health equity is embedded in delivery has not been assessed.

**Objectives:**

We used an equity assessment tool to examine our regional health protection acute response service.

**Methods:**

We synthesised evidence on inequalities in frequency and severity of twenty-one priority hazards, and inequity in at-risk groups' potential ability to access and benefit from health protection acute response functions. We mapped our findings against the UKHSA Acute Response Service Specification to identify functions requiring equity-focused tailoring. Strengths and weaknesses of current policies, guidance and resources were summarised using a published framework for equity-oriented public health action.

**Results:**

Inequalities in hazard frequency were identified for all hazards, and in severity for 17/21 (81%). Inclusion health groups, people living in deprivation, minority ethnic groups, gay and bisexual men, extremes of age, and those in congregate settings were most affected. All assessed populations had potential inequities in accessing or benefitting from interventions. Twelve of fifteen (80%) acute response functions showed overlap between disproportionately affected groups and those facing potential inequities in access or benefit. Strengths included tailored guidance and specialist advice; weaknesses included inconsistent consideration of communication and accessibility needs, limited equity data, and gaps in systematic equity-focused processes.

**Conclusions:**

Health protection hazards consistently demonstrate avoidable inequalities. While existing actions address disparities, gaps identified in systems, policies and practice limit equitable delivery of acute response services. Strengthening workforce capability, data and evaluation, clinical governance, policies and procedures, and use of community insight will help embed equity across acute health protection functions.

